# Assessing higher-order visual functions: seeing beyond a flat earth

**DOI:** 10.1590/1980-5764-DN-2024-0206

**Published:** 2025-05-19

**Authors:** Henrique Soares Dutra Oliveira, Danielle Calil de Sousa, Sarah Teixeira Camargos, Francisco Cardoso, Paulo Caramelli

**Affiliations:** 1 Ciências Médicas Minas Gerais, Faculdade de Medicina, Departamento de Neurogeriatria, Belo Horizonte MG, Brazil.; 2 Universidade Federal de Minas Gerais, Faculdade de Medicina, Departamento de Neurologia, Belo Horizonte MG, Brazil.

**Keywords:** Vision Disorders, Color Vision Defects, Visual Perception, Spatial Processing, Agnosia, Transtornos da Visão, Defeitos da Visão Cromática, Percepção Visual, Processamento Espacial, Agnosia

## Abstract

Vision is a sensory resource that enables the creation of an internal model for perceiving the external world. The act of seeing is much more than the mere process of light reflection on the retina. Higher-order visual areas play a fundamental role in the ability to see, providing visual perception and object recognition functions that are resources beyond the process of perceiving color, motion, and form. Disorders of higher visual abilities can be the result of a variety of etiologies. The bedside assessment of higher visual cortical functions provides clinicians with a valuable tool when suspecting individuals with these conditions.

## RESUMO.

A visão é um recurso sensorial que possibilita a criação de um modelo interno de percepção do mundo externo. Enxergar é muito além de ver. Áreas visuais corticais superiores desempenham um papel fundamental na habilidade de enxergar, visto que proporcionam funções de percepção visual e de reconhecimento de objetos que são recursos além do processo de perceber cores, movimento e formas. Desordens das habilidades visuais superiores podem ser fruto de diversas etiologias. Conhecer habilidades à beira leito das funções corticais visuais superiores permite agregar um novo arsenal ao clínico durante a suspeita de indivíduos acometidos com essas condições.

## INTRODUCTION

Humans are visual creatures. It is estimated that approximately 35% of the brain cortex is dedicated to processing visual information^1^. How we transform constantly changing and complex visual information into a coherent three-dimensional (3D) perception is not a one-man job. Anatomical and physiological studies of the monkey visual cortex anticipated that the human visual system would be consistent with a variety of different paths, each specialized for a particular visual attribute^2^. Visual processing involves signals relaying from retinal ganglion cells via the lateral geniculate nucleus to the striate cortex. In the visual cortex itself, there is initially strong retinotopic localization such that lesions in that area can cause a specific visual defect with a macula sparing^3^. The extrastriate visual cortex is highly organized by a process with different areas involved in color, motion, and shape perception. This process occurs along a core synaptic hierarchy that includes primary sensory, upstream unimodal, downstream unimodal, and heteromodal zones of the associative cortex^4^. One of the most accepted propositions––the dual theory of two visual systems––states that extrastriate regions group into two streams: the ventral occipitotemporal ("what"), which is involved in object recognition, and the dorsal occipitotemporal ("where"), which is related to spatial processing^5^. Nevertheless, the guiding theory is now being reconsidered since evidence for contributions from the ventral stream system to the dorsal stream system plays a crucial role in mediating complex and flexible visuomotor skills, and complementary evidence points to a role for the dorsal stream in certain aspects of 3D perceptual functions in the ventral stream^6^. Refraining from theoretical discussions, it is still clinically relevant to recognize dysfunction in the process of visual information^7^. Clinicians who consider the neuro-ophthalmological examination to include only the assessment of the vision (visual acuity and visual fields), the eyes, and the ocular adnexa^8^ may fail to identify high-order diseases of the visual cortex. This review focuses on a bedside practical approach for assessing high-order visual functions, incorporating some caveats and potential pitfalls for the busy clinician.

### Theoretical basis

One of the main functions of the visual system is building an internal model or a percept of the external world: a general-purpose representation of the exterior world. It consists of a set of relatively independent input–output lines, each responsible for the visual control of a particular class of motor outputs^9^.

**Figure 1:**
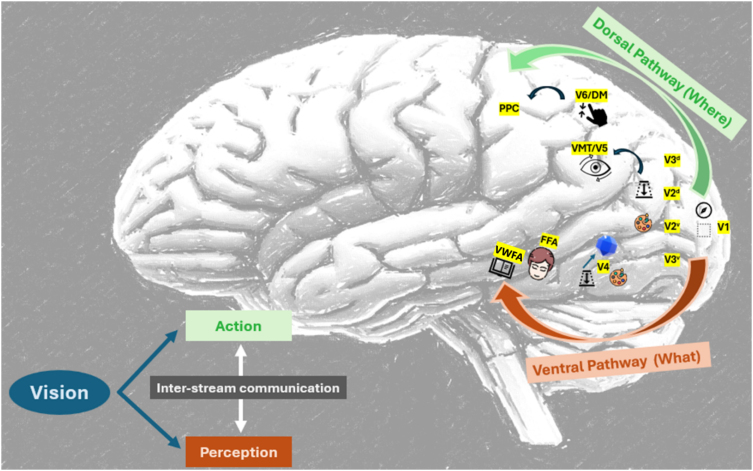
A simplified depiction of the visual processing-related striate and extrastriate cortical areas.

Source: Adapted from Prasad and Dinkin^13^.

The rigorous retinotopic arrangement of the primary visual cortex's inputs, which perfectly corresponds to spatial locations in the contralateral homonymous visual field, is one of its key characteristics. Because the neurons in the primary visual cortex are selective for particular orientations of luminance contrast, these neurons’ response characteristics allow them to recognize the edges of an object in visual perception^10,11^. Furthermore, the primary visual cortex is where the early processing of color composition, brightness, and motion direction takes place^12^. Many nearby cortical areas continue to analyze particular features of visual information after initial processing in the main visual cortex. These regions are designated as V2, V3, V4, V5, lateral occipital area, and fusiform face area (FFA) (Figure 1), and are found in the occipital, temporal, and parietal lobes^13^. Higher-order visual areas respond more consistently to changes in viewing conditions than the retinotopic structure in the primary visual cortex, where responses are highly impacted by factors, such as object position, orientation, and lighting within the visual field^14^. They do not follow the same rigid point-to-point retinotopic layout as previously noted^15^.

In a nutshell, vision in humans serves two distinct yet interconnected functions:

- Perceiving objects and their relationships, which forms the basis for the organism's cognitive processes (the ventral stream);
- Controlling actions aimed at or in relation to those objects (the dorsal stream), involving the programming and real-time guidance of specific motor outputs.

According to Goodale and Milner, the ventral stream plays a major role in constructing the perceptual representation of the world and the objects within it, while the dorsal stream mediates the visual control of actions directed at those objects^16^. Lesions in the former tend to cause problems, such as central hemiachromatopsia, visual agnosia, alexia without agraphia, and prosopagnosia. Dysfunctions in the latter could cause hemineglect, optic ataxia, oculomotor apraxia, and simultagnosia. Deficits can also be categorized into levels of visual processing from low-level (e.g., processing in V1/V2/V3) to high-level (e.g., processing in the inferior parietal lobule and the lingual gyrus). Figure 1 summarizes the disruption of the extrastriate regions of the cerebral cortex responsible for higher visual processing.

The extrastriate cortex's successively specialized sections (V1–V6) receive visual inputs in an anterior fashion. Either the ventral or dorsal stream is thought to include these extrastriate cortical regions. The striate cortex, or V1, is located posteriorly and is where the optic radiations send their raw visual data. At this level, the fundamental location and orientation of a visual stimulus's borders are encoded. Ventral V2 (V2v), the first segment of the ventral stream, starts to process the aspects of visual object identity (the "what" stream), starting with color analysis and a foreground/background (depth) analysis of visual stimuli. Additional processing takes place in ventral V3 (V3v) and continues in V4, which is crucial for distinguishing between colors and has neurons with response frequencies that are tunable to various light wavelengths. At this level, basic geometric shapes are also encoded. To facilitate effective facial recognition, information is sent anteriorly to the inferotemporal cortex, home of the FFA, which encodes facial traits. The fusiform gyrus is home to the visual word form area (VWFA), which can cause pure alexia on its own without any accompanying hemianopia. In dorsal V2 (V2d) and dorsal V3 (V3d), visuospatial processing follows the dorsal stream, or "where" pathway, which feeds anteriorly to the mesiotemporal visual area (MT/V5), which is crucial for motion perception. The dorsomedial cortex (V6/DM) aids in the guidance of reaching and other visually guided motor activities in response to the visual inputs related to self-motion. The posterior parietal cortex (PPC) is where additional visuospatial processing takes place. Numerous factors are involved in spatial processing itself, such as the task's objective and the frame of reference being used. Multiple egocentric reference frames (with respect to body parts) and reference frames related to objects or environments can be used in spatial tasks. These many reference frames have various functions in the sense of space. While the inferior part, which is located near the temporal lobe, mediates other spatial processing utilizing numerous frames of reference, the superior portion mediates visually guided actions.

## FRAMES OF REFERENCE

### Ventral stream

#### Central hemiachromatopsia

Cerebral achromatopsia is defined as a total or partial loss of color vision. Almost every documented incidence of cerebral achromatopsia involves bilateral damage (usually a stroke) to a relatively narrow region of the cortex: the inferior surface of the temporo-occipital regions, namely, the lingual and posterior fusiform gyri^17^. Unless the patient is specifically evaluated, a unilateral lesion causing hemiachromatopsia frequently goes undetected^18^. Sometimes, the term dyschromatopsia is favored if the deficit is partial. Achromatopsia subjects say that everything looks grayscale^19^. Color discrimination impairment can impact tasks like differentiating currency, stamps, and traffic signals. Frequently they go along with a homonymous superior quadrantanopia^20^.

Achromatopsic patients often see brightness normally, but their ability to distinguish between hue and saturation is impaired^21^. These individuals can employ their typical hemifield when being evaluated: the finest way to demonstrate their impairment is to move a colored item along the vertical meridian such that when it transitions from the defective to the normal field, the color will stand out^20^.

As depicted in Figure 1, the inferior occipital cortex (V4) is an important area in the cortical region in humans that is specialized for color processing^22^. Apart from hemiachromatopsia resulting from a lesion in region V4, there are other color processing-related brain diseases. Patients with color anomia, a categorical language disease, are specifically impaired in naming colors, but they are able to name other kinds of items^23^. With this disease, color perception is unaffected; yet, performance on a color-matching test is normal despite incorrect color identification. Another phenomenon known as color agnosia is ill-defined and has been construed in a number of ways^24,25^. The most widely accepted explanation is that it alludes to compromised semantic knowledge of colors even in the presence of intact color vision (e.g., failing to recognize that a taxi is yellow, or a lime is green)^13^.

#### Visual agnosia

Visual agnosia may be defined as the failure to recognize visually presented materials despite adequate visual acuity, visual fields, visual scanning, language function, and general mental ability^26^. Perception encompasses various levels of internal representation, spanning from basic sensory images to higher-level abstract representations of real objects like faces or complex sensory experiences. Each sensory modality is organized hierarchically and in parallel, featuring both feed-forward and feedback connections among multiple representations within the sensory domain. The recognition of sensory stimuli relies on modality-specific association cortices. For each sensory modality, there exist distinct yet overlapping networks responsible for the different aspects of sensory stimulus recognition and categorization. Dysfunction or lesions in different neural components of these networks can result in different forms of agnosia, specific to each sensory modality^4^.

Individuals with visual agnosia are unable to identify by sight things that they would have been familiar with in the past^27^. They are unable to name the percept or provide distinct semantic identifying information. Visual agnosia is further separated into two categories: associative visual agnosia, in which high-level perception is preserved but the percept is unable to activate semantic identifying information (usually the anterior left temporal lobe), and apperceptive visual agnosia, where a deficit in high-level perception is implicated (usually widespread, bilateral occipitotemporal infarction). Heinrich Lissauer was the one who first presented this proposition in 1989^28^.

According to Milner and Teuber^29^, a patient with associative visual agnosia experiences a visual perception that is "stripped of its meaning." Patients with associative visual agnosia may describe an object's look, sketch it accurately, and distinguish it from other examples, but cannot specify its name or purpose^30^. The loss of object recognition is limited to visual perception alone; patients may answer factual questions about things and recognize them when questioned using other sensory modalities, such as touch or hearing.

Defining the borders of a visual object is an important step in efficiently managing vast amounts of visual information. Without the ability to segment an image into regions corresponding to objects, humans would be unable to comprehend complicated visual inputs and would futilely attempt to distinguish non-objects made up of overlapping or juxtaposed items. Region V2 contributes to picture segmentation by modifying the inputs received from region V1. Image segmentation at this step is heavily influenced not just by objective bottom-up inputs, but also by internal top-down mechanisms that represent the current perceptual interpretation of the visual signal^13^.

#### Prosopagnosia

This is the incapacity to identify known faces^31^. Humans are highly skilled at deriving facial features from which they may quickly and reliably recognize a face. Even at 30 min old, infants follow a moving face more closely than they do other moving patterns with the same contrast^32^. Despite a familiar face's wide range of expressions and orientations, we can identify it virtually quickly.

Face processing disorders represent a "family of disorders"^33^ with several components. In order to process faces, we must use our "memory for faces" to determine whether or not we have seen someone before, integrate the many aspects into a coherent sense (age, sex, and height), and decipher emotional facial expressions in order to react properly.

Lesions of the right-lateralized posterior network are the cause of the majority of specific kinds of acquired prosopagnosia^34^. Patients with left temporo-occipital injuries may experience difficulties with face identification. Some authors were able to identify and label the FFA, which is located in the medial aspect of the inferior occipitotemporal region of the right fusiform gyrus, using functional magnetic resonance imaging (MRI) while patients saw photographs of faces and other non-facial objects^35^.

Additionally, faces are the only objects that the posterior superior temporal sulcus selects for^36^. The amygdala, the paracingulate gyrus, portions of the inferior frontal gyrus, and more anterior sections of the superior temporal sulcus are additional areas that might be involved in face perception^37^. Instead of just facial recognition, it is probable that some of these areas are active when doing activities that demand a high level of visual recognition. It is worth mentioning another peculiar phenomenon that besides typically associated with psychotic disorders could also be secondary to organic causes: the "misidentification syndromes"^38^. Disorders such as Frégoli syndrome and Pick's reduplicative paramnesia, respectively the delusional belief that a familiar person has repeatedly changed their appearance becoming them a double and the subjective belief that a place has been duplicated and exists simultaneously in two or more locations, have been associated with lesions in the anterior part of the right fusiform gyrus and a smaller area in the nearby anterior, middle, and inferior temporal gyri; in these cases, these conditions are believed to emerge by the disruption of the connections of these highly specialized areas with the most anterior, inferior, and medial parts of the right temporal lobe where long-term memory and mechanisms for the retrieval of information that are required for the recognition of faces and scenes are stored^39^.

#### Alexia without agraphia (pure alexia)

Alexia without agraphia is the term used to describe the loss of reading comprehension while preserving writing abilities. Dejerine was the first to speculate that the left angular gyrus stored the visual representations of words needed for reading and spelling when he introduced the term in 1892^40^. He claimed that disconnecting both hemispheres’ visual inputs from this region (but leaving the area and neighboring language zones intact) would damage reading while preserving writing. Writing does not, of course, rely entirely on visual information. This disconnection is usually caused by the combination of a left hemisphere lesion causing a right visual field defect (typically homonymous hemianopia) and the extension of the lesion into the splenium of the corpus callosum, which blocks visual information from the left visual field as it crosses between hemispheres^41^.

In contrast to Dejerine's work, it is no longer considered that the inferior temporal structures damaged in pure alexia passively transmit visual information to the left angular gyrus and other language centers. Instead, individuals are actively processing the visual form as they go along the left temporal "what" stream. The VWFA, located in the left lateral occipitotemporal sulcus and constituting a posterior section of the left fusiform gyrus, is dedicated to the processing of letter strings^42^. The VWFA is exclusive to the left hemisphere. Visual information provided to the left hemisphere must pass via the splenium of the corpus callosum before reaching the left angular gyrus. In individuals with substantial callosal injury, the VWFA is triggered only by stimuli present in the right visual field, resulting in hemialexia, which includes reading problems at the beginning of words^43^.

Pure alexia can also develop when injury to left fusiform structures, such as the VWFA^20^, causes selective visual agnosia but no corpus callosum lesion. According to Geschwind^44^, lesions in the association cortex, which connects cortical regions within one hemisphere, nonetheless result in a disconnection since visual information cannot be transmitted to the left angular gyrus. Pure alexia can develop without a cortical lesion, such as when a lateral geniculate nucleus lesion is paired with a splenic lesion (producing right hemianopia)^45,46^. Because of the intimate connections between the color center and the VWFA, patients with pure alexia frequently experience achromatopsia and/or color anomia. Reading involves multiple processes, including eye focus and saccadic motions.

### Dorsal Stream

#### Optic ataxia

Misreaching visual targets that cannot be attributed to weakness, clumsiness, or incoordination is a characteristic of the disease known as optic ataxia^47^. In the most evident form, individuals suffering from this condition are able to describe an object that is clearly visible to them, but when trying to pick it up, they miss the target and are left to haphazardly search for the thing^48^. A patient may be unable to reach a set of pictured keys if their condition is limited to the visual modality; but, if the keys are rattled while they have their eyes closed, they may be able to reach them appropriately.

One of the factors that can influence performance in optic ataxia is the hand used to reach and the hemispace in which the object is presented^49^. Some individuals only make mistakes with one hand, whereas others only make mistakes in one hemispace, or side of the environment, when using both hands. Other patients only make mistakes when reaching with one hand in one hemispace. Therefore, only when reaching with the left hand patients in this situation miss a target in the left hemispace.

Although disorders sometimes co-occur, optic ataxia and ocular apraxia (see description below) are localized in different areas of the posterior parietal brain. Optic ataxia is caused by damage to the pathways linking the parietal reach region and the medial interparietal area to the dorsal premotor cortex^50^. Dorsal parietal area 5 appears to have a role in the online control of reaching, which means that it uses proprioceptive and visual feedback to help rectify reaching errors made during an action^51,52^. Damage to the anterior interparietal area and its connections to the ventral premotor cortex appear to be the cause of difficulty gripping items.

#### Simultanagnosia

"To see but not two see" may be one of the most precise characteristics of simultanagnosia: a condition of visual perception which is defined by the preservation of single-object recognition but an inability to understand complex visual arrays^53^. The perception of local elements of a scene is intact, but subjects have the inability to perceive its global elements: "missing the forest for the trees"^54^.

Bilateral posterior lesions have been observed in the majority of cases, including the exquisitely researched patients of Holmes^55^. Conversely, cases of simultaneous agnosia in individuals with unilateral left occipital lesions have also been documented^56^. Certain authors^48^ contend that perception is influenced by top-down factors, including the significance of a stimulus. When presented with an image of a hand, patients are more likely to report seeing a hand than a finger or knuckle. This is because most of the time, patients report the largest relevant "object" in an array. The letter strings follow the same principle. Patients’ performance with non-words, or unfamiliar letter strings for which there is no stored knowledge to aid identification, clearly demonstrates the impact of meaningfulness or familiarity.

There are various theories as to why simultanagnosia occurs. According to some researchers, "sticky" visual attention—that is, when presented with an array, patients recognize the first item that catches their attention but are unable to move it away from the objects and instead get fixated on them—is the source of simultanagnosia^57^. To others, the condition is caused by a breakdown in the binding of information between object identity and position calculations made in the ventral or what stream and the dorsal visual stream^58^. The specifics of the disorder may reflect a range of higher-level visual processing abnormalities, the exact form of which depends on the location, kind, and amount of the pathology.

#### Ocular apraxia

Apraxia is the inability to perform skilled actions and to use instruments in a proper manner that cannot be explained by weakness, incoordination, major sensorimotor defects, or cognitive impairment. The term apraxia is sometimes used to describe a variety of problems that have little or nothing to do with skilled actions, such as oculomotor apraxia^59^. Holmes (1918) was the first to use this denomination to characterize five patients who were able to accurately direct their eyes to a sound or a region that the practitioner verbally described, but not to a visually presented target^55^. Without real paralysis, the significant disruption of the onset of purposeful saccades in all directions could be classified as acquired ocular motor apraxia^60^. Saccade initiation may require at least two parallel excitatory suprareticular pathways^61,62^. Their sources are the superior colliculus and the frontal eye field, respectively, from which they project directly onto the brainstem's premotor reticular structures. Lesion experiments on monkeys^63^ provide support for this organization: unilateral damage to the frontal eye field or the superior colliculus alone caused only a transient disruption of saccades, whereas bilateral damage to both structures caused a severe and permanent impairment of saccades. Additionally, it has also been postulated that the posterior part of the lateral bank of the intraparietal sulcus, which is housed in the inferior parietal lobule, may actually be the source of an excitatory channel that the superior colliculus relays in^64^. Since only bilateral frontoparietal lesions markedly impair saccades beginning in brain hemisphere pathology, it is possible that the actions of the frontal and parietal areas overlap slightly in a normal person^65^.

#### Balint syndrome

The three conditions that constitute Balint syndrome are ocular apraxia, optic ataxia, and simultanagnosia. The Hungarian physician Rudolph Balint documented in 1909^66^ an engineer whose visual acuity, strength, and dexterity were normal but whose significant inability to change spatial focus prevented him from assembling models. He was discovered to have bilateral parietal infarcts at postmortem.

It is interesting to note that Wolpert's case description from 1924^56^ was the first to formally identify simultanagnosia as a feature of Balint syndrome. He depicted that the patient saw "only details that he could not sum up" and was unable to identify the action depicted in the image when he saw the patient attempting to describe the events in a scene.

It is typically caused by bilateral parietal lesions, leading to a significant disturbance of visuospatial attentional systems. Even when basic visual functions like acuity and ventral stream functions like object recognition are preserved, patients suffer greatly from the inability to disengage and focus on different areas of a visual scene.

#### Hemispatial neglect

One can distinguish the right and left sides (hemispaces) of the environment around them. Neglect is a condition in which attention is no longer focused on the self, or a particular bodily part, or on outside things due to deficiencies in visual attention^27^. Almost usually, the left hemifield is neglected. This can be explained by the fact that the right hemisphere keeps an eye on both hemispaces, whereas the left hemisphere watches over the right hemispace. Neglect does not arise when there is a left hemisphere lesion because the right hemisphere can still scan the whole visual field. In contrast, in the event of a right hemisphere injury, the left hemisphere is exclusively responsible for monitoring the right hemispace; hence, left-sided neglect arises due to a deficiency in left hemispace monitoring^67^.

At least three unique frames of reference, focused on the body midline, head midline, and visual field, may be used to distinguish between the right and left hemispheres^59^. The three hemispaces are aligned in the conventional anatomic position, with the head and eyes pointing forward; they are dissociated in numerous activities. Patients may differ in the coordinates used to define left hemispatial neglect. Egocentric coordinates, which are mostly oriented on the patient's body, head, or eyes, maybe where it happens. Regardless of whether an object is on the patient's right or left side, the coordinates of that object may be used to determine the left neglect in other patients^13^.

There is robust evidence that neglect can be influenced by hemispace, which is defined by all three coordinate frames. Previous research has shown that the location of the stimulus to be bisected with regard to the body midline affects phenomena like line bisection, with performance being better on the right side of the body than the left^68^. Furthermore, the positioning of the hands during testing may have an impact on tactile extinction: individuals with neglect may exhibit reduced neglect of the left hand when both hands are positioned on the right side of the head or torso.

#### Topographagnosia

The inability to find one's way into a familiar environment and to learn new routes is what characterizes this condition^69^. Navigation involves numerous steps, as with other complex activities, and multiple solutions can be applied depending on the situation. A number of topographagnosia types have been described, such as heading disorientation, which is a failure to discern the relationship between objects in the environment; impaired cognitive map formation, which is the inability to form a mental layout of scenes; and landmark agnosia, which is the inability to recognize buildings and scenes^20^. The right temporal lobe, and more specifically the right hippocampus, is the primary lesion location associated with landmark agnosia^70^.

#### Riddoch syndrome

The capacity to recognize motion in an otherwise blind visual field is known as Riddoch syndrome^71^. This condition, known as statokinetic dissociation, occurs when an object is only seen to be there when it is in motion. Riddoch^72^ first described this phenomenon in 10 British World War I soldiers who had been injured from penetrating bullets affecting the occipital lobe. Some argue^13^ that the Brodie helmet, which had the shape of a soup bowl, did not confer much protection to the base of the head, leaving this region more vulnerable to lesions.

V5, the cortical region in the occipital lobe dedicated to motion processing, is located dorsal to the main visual cortex. Regarding how visual inputs reach this region and cause the statokinetic dissociation observed in Riddoch syndrome, there is still some debate. When functional MRI responses in a patient with this disease were not detected in region V1, some researchers hypothesized the existence of extra direct subcortical connections to area V5^73,74^. Other evidence converging to this hypothesis comes from intact structural connections between the lateral geniculate nucleus and V5, as shown by tractography^75^ in patients. Animal studies^76^ on monkeys also demonstrate that the ventrolateral pulvinar and the superior colliculus pathways play an important role in the preservation of motion detection in blindsight through direct communication with extrastriate areas, including V5. Conversely, the clinical phenomenology of Riddoch syndrome may not always depend on direct inputs to area V5 that are spared. In the lesioned portion of the primary visual cortex, other researchers^77^ have discovered small islands of activation. These spared areas may not support aspects of vision processing like shape, color, or form, but they might effectively relay information to V5 that is sufficient for motion processing.

## CLINICAL ASSESSMENT

The precise diagnosis of a high-order visual dysfunction is particularly difficult since patients find it difficult to express their symptoms. Compared to visual symptoms resulting from lesions in the ocular or pre-cortical afferent visual pathway or associated binocular visual dysfunction, the chief complaints are frequently ambiguous or are lacking in specifics. Often, before a proper diagnosis is obtained, patients first visit an ophthalmologist to look for the ocular or refractive causes of their higher-order visual dysfunction^78^.

The clinician should start looking for signs of higher-order visual dysfunction when there are normal visual acuity, normal ocular and binocular exams, and fairly ill-defined visual symptoms linked to reduced functional tasks. One of the most prevalent visual complaints in this context is trouble reading^78^. Other common complaints are: "My glasses don't function as well as they used to," "Seeing is becoming more difficult," "I can't see clearly," "It's difficult to read road sign when driving because they appear so quickly."

Having in mind a structured approach to these disorders is essential and asking the right questions could help the clinician grasp some important clinical clues to the diagnosis^79^. Inquire the patient if they have problems reading scrolling text on a screening. Do they struggle reading cursive or certain types or styles of font? Do they have any problems with parking or driving lately? Do they find it difficult to identify objects or faces? Do they experience any difficulties perceiving depth? Table 1 summarizes the clinical presentation, the bedside assessment, and neuropsychological tests regarding the most common syndromes related to the ventral and the dorsal visual streams.

**Table 1:** Higher cortical visual syndromes: Pearls and oysters.

Visual agnosia	Cerebral achromatopsia	Alexia without agrafia	Prosopagnosia
**Clinical Presentation**	**Clinical Presentation**	**Clinical Presentation**	**Clinical Presentation**
It will be noted if the patient is unclear or reluctant to use an object, or that it perplexes them. This contrasts with limb-kinetic apraxia, which is the inability to perform a movement required to use an object or technology even while the person recognizes it and knows why it is there.	Achromatopsia is limited to the contralateral visual hemifield if cerebral impairment is limited to one side of the brain inside the ventral occipitotemporal area. Patients rarely complain of their vision becoming boring, especially if they work in or enjoy activities related to the creative industries. The issue is typically found during the exam.	Reading difficulty, which could be said to be worse when attempting to read material written in cursive or using font types. The patient will claim that they are unable to read their own handwriting due to this deficiency, which also affects their ability to read what they have written. Patients read extremely slowly and make numerous errors. They may try to compensate by using letter-by-letter reading but frequently confuse similar looking letters.	Rather than having trouble recognizing faces, some patients describe this as forgetting a name. Inquiries should be made further to distinguish between poor facial recognition and name forgetfulness. Can the patient, for example, remember the name if the individual speaks? If so, they might be able to identify someone based only on the auditory processing of their voice, rather than visual processing of their face features.
**Bedside Assessment**	**Bedside Assessment**	**Bedside Assessment**	**Bedside Assessment**
Object naming and the capacity to explain the meaning of unidentified objects. Ask the patient to sketch the object or replicate a drawing. Requests the patient to describe and demonstrate what they see - holding the object would be consistent with a visual agnosia rather than a loss of semantic understanding if it enables the patient to recognize the object where vision has not.	■ Use eye drop colored caps during confrontation visual field testing; ■ Ishihara plates. 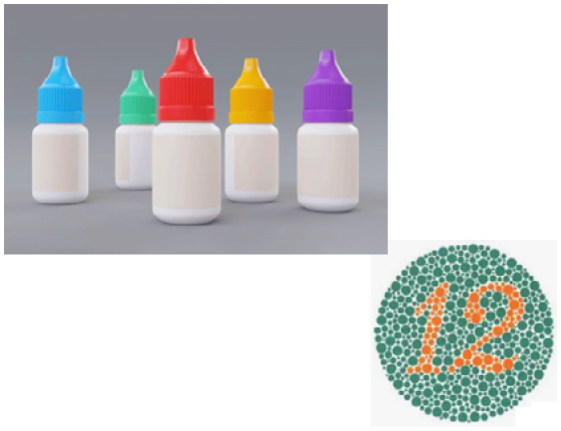	Ask the patient to write a sentence of their choosing and then ask them to read what they wrote aloud after some time.	■ Famous people recognition 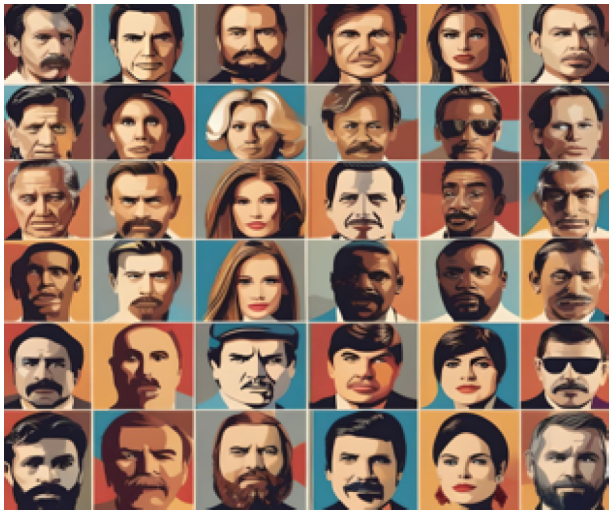
**Neuropsychological Test**	**Neuropsychological Test**	**Neuropsychological Test**	**Neuropsychological Test**
■ ACE; BORB; CORVIST; VOSP.	■ Farnsworth Munsell hue test;Hue test form CORVIST.	■ ACE; CORVIST.	■ CFMT; FFT; RMF; BFRT.
Ocular Apraxia	Optic Ataxia	Riddoch Syndrome	Simultagnosia
**Clinical Presentation**	**Clinical Presentation**	**Clinical Presentation**	**Clinical Presentation**
Difficulty performing visually tasks like reading and driving. Spouses or family members may report that patients have more severe oculomotor apraxia if they are unable to focus the patient's visual attention or eyes on anything that is being pointed out or indicated.	Usually, a spouse or other family member reports this later on in the treatment. They may mention that the patient stumbles or does not respond when someone approaches to shake hands with them. Having trouble reaching for objects or pouring liquids into glasses is another noteworthy report.	Individuals suffering from Riddoch syndrome are unable to perceive color and form in objects due to a visual field impairment. If an object is motionless, the patient is not aware of it. If the object moves, on the other hand, it can be sensed with some degree of reliability, though not with a correct sense of color or form. The ability to discern motion characteristics like direction and speed can be a part of preserved motion discrimination.	Cannot find things that are later noted to be in plain sight (can also be a symptom of visual crowding). Can impact driving. The struggle recognizing several components of a complex visual situation.
**Bedside Assessment**	**Bedside Assessment**	**Bedside Assessment**	**Bedside Assessment**
Examine fast eye movements (i.e., saccades) to visual targets (test all four quadrants of the visual field). Patients exhibit failure in the quick phase of optokinetic and vestibular nystagmus resulting in "locking up. In this phenomenon, the patient thrust their heads first when performing saccade examination.	Examine ability to reach and grasp a visual target. Ask patient to look at the examiner's nose while attempting to reach to the examiner's finger that is held peripherally. Subjects with optic ataxia miss the target. Different from cerebellar ataxia optic ataxia these subjects do not struggle when reaching for their own's nose.	Use moving fingers when performing confrontation visual field testing 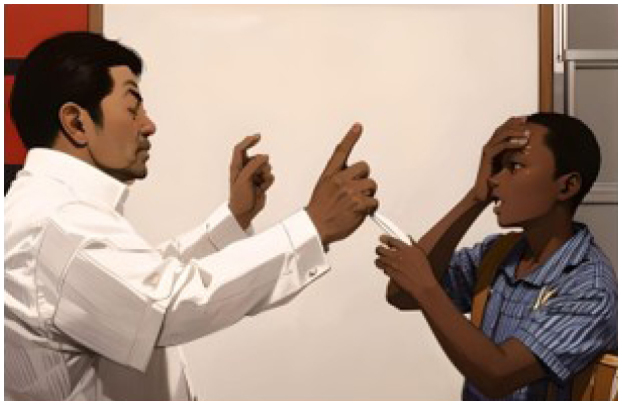	■ Navon figure (can´t make sense of letter "Xs" – just sees the "H"). 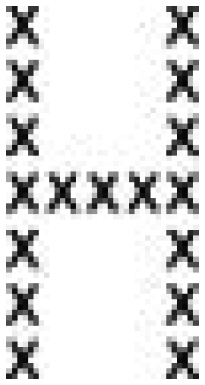
**Neuropsychological Test**	**Neuropsychological Test**	**Neuropsychological Test**	**Neuropsychological Test**
■ Not Applicable	■ Not Applicable.	■ Not Applicable.	■ BPT; The Cookie Theft Picture - Boston Diagnostic Aphasia Examination.
Hemispacial Neglect	Impaired Depth Perception	Topographagnosia	Visual Crowding
**Clinical Presentation**	**Clinical Presentation**	**Clinical Presentation**	**Clinical Presentation**
Individuals may not shave, apply makeup, or use a comb on the left side of their face. They may also disregard the left side of their body. Even after being shown their left arm, patients who have experienced personal neglect frequently refuse to acknowledge that they own it. It's possible that they won't investigate and explore the contralateral hemibody and even deny any weakness when its present.	Inadequate sense of depth (usually mentioned out loud). Patients are typically able to provide examples, such as misjudging the depth of a curb or another variation on a walking path. Mistakes in depth when parking a car are frequently reported in the early stages of the course, when patients are still operating a vehicle.	Reduced ability to see scrolling text on television, as well as discrete difficulties following action in movies or watching things in motion, such football games on television. Concerns regarding poor motion and/or spatial processing should be raised in light of reports of patients being confused in situations that should be simple for them to self-navigate, such as grocery stores or hotels.	The inability to distinguish details when two or more visual stimuli are present. Standard report: Finding items difficult, especially notably when looking about in a cluttered area (in a refrigerator, a drawer, or on a cluttered dining room table, for example). Number "seeing" issues, such as mistaking the number 5050 for the number 500, might arise. Crowding depends on the eccentricity of a target object and how densely spaced the surrounding objects – Bouma factor.
**Bedside Assessment**	**Bedside Assessment**	**Bedside Assessment**	**Bedside Assessment**
Drawing (clock) and line cancellation exercises. On examination room patient should be asked to point to and/or name all the objects that they could see on both sides. Sensory inattention and visual neglect may be assessed clinically with unilateral and separately stimulus and after with simultaneously stimulus.	■ Horizontal Lang two-pencil test. 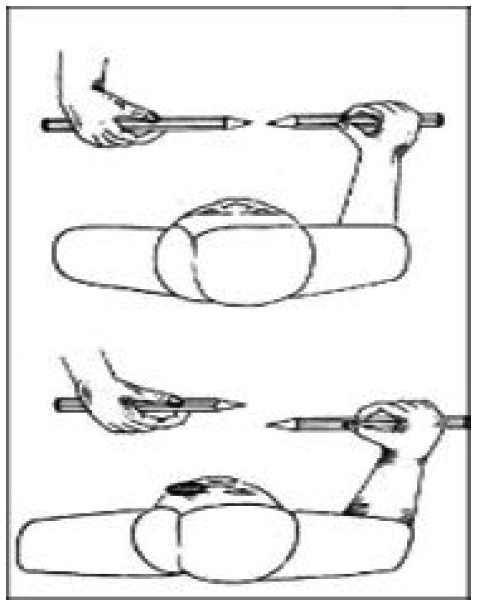	Rarely possible to test in an office setting; one option is to take the patient on a short route, and after a brief pause, ask the patient to find their way back to the exam room using the same route in reverse.	■ Lea Symbols; ■ Kay Pictures.
**Neuropsychological Test**	**Neuropsychological Test**	**Neuropsychological Test**	**Neuropsychological Test**
■ RBIT.	■ Not Applicable.	■ Not Applicable.	■ CORVIST; CCC.

Abbreviations: ACE, Addenbrooke's Cognitive Examination; BORB, Birmingham Object Recognition Battery; BPT, the Birthday Party Test; BFRT: Benton Facial Recognition Test; CCC, Cambridge Crowding Cards; CFMT, Cambridge Face Memory Test; CORVIST, Cortical Vision Screening Test; FMT, Famous Faces Test; RBIT, Rivermead Behavioral Inattention Test; VOSP, Visual Object and Space Perception Battery; RMF, warrington Recognition Memory for Faces.

As it is frequent in neurodegenerative disorders, a patient with a known moderate or severe dementia, who is presenting for a visual evaluation, may not be able to provide a full history or provide insights into visual perception issues due to anosognosia. In these situations, a partner providing care may notice that the patient is not reading as much or appears to be having trouble with visual tasks. Accompanying impairments in posterior cortical functions may include dyscalculia or apraxia, and a thorough examination may discover these symptoms. The examiner must ask about using the TV remote control, wearing a button t-shirt, or opening a locked door with a key, if there is a suspicion of apraxia. Patients must be inquired about calculating a supermarket shop bill without using a cell phone to identify the symptoms of dyscalculia. Ultimately, additional testing and inspection are required because it can be challenging to diagnose apraxia and dyscalculia based on past records.

## PITFALLS: VISUAL PERCEPTUAL DISORDERS AND VISUAL ANOSOGNOSIA––CHARLES BONNET SYNDROME AND ANTON SYNDROME

A visual perceptual disorder may be regarded as a divergence from the normal sense of "seeing" or, to put it another way, the conscious, visual experience of the world we get when we open our eyes^80^. They can be "positive" (such as hallucinations) or "negative" (such as visual agnosia, which is characterized by a "visual perception that is deprived of its meaning")^81^. One distinguished condition, Charles Bonnet syndrome, describes the phenomenon of vivid visual hallucinations attributable to visual loss or visual deprivation, occurring without voluntary control. These hallucinations may occur independently as a consequence of visual impairment, with or without additional neuropsychiatric factors^82^. Typical phenomenology ranges from unformed shapes and irregular patterns to more complex formed images comprising animals, people, and life-like scenes^83^. Given their wide range of visual symptoms, it was not until lately that a unifying classification managed to place this heterogeneous group under one roof through a mechanistic approach. The idea is based on a "hodotopic framework" which consists of specialized cortical subregions (topology) and their connections (hodology)^84^. The hodotopic hypothesis conceptualizes that the cortical functional epicenter, or "topic organization," inside the central nervous system forms an integrated, vast, plastic network that is linked by both short- and long-scale white matter fibers, or "hodological organization." It postulates that parallel streams of information within an interactive, multimodal, broadly distributed circuit are dynamically modified and lead to brain activity^84,85^. According to the hodotopic model, an extension into medial white matter also results in visual hypoemotionality (a deficit of visually evoked emotions with preserved emotional responses to non-visual stimuli^86^) or visual amnesia (a deficit of registering novel visual experiences in short-term memory with the preserved ability to register non-visual images^87^). A ventral or lateral temporal cortical lesion leads to specific deficits related to the cortical specializations lost. Specific visual attribute hallucinations are linked to the hyperfunction of particular visual cortex areas. Uncertainty surrounds the effects of hyperconnectivity on the indirect or direct occipitotemporal pathways^81,88^; however, it may be the cause of symptoms like exaggerated emotional reactions to visual stimuli and synesthesia between visual features. Ffytche and Howard^81^ proposed an eight-category classification of visual hallucinations, consisting of the following: tessellopsia; hyperchromatopsia; prosopometamorphopsia; dendropsia; preservation; illusory visual spread; polyopia; and micro/macropsia. Table 2 summarizes the most common visual perceptual disorders. Moreover, another important condition to bear in mind is Anton syndrome, which refers to blindness due to bilateral occipital lesions with a lack of awareness (anosognosia) of the deficit. The term was coined by the Austrian neuropsychiatrist Gabriel Anton in 1899^89^. The precise neuroanatomic basis for the loss of awareness of the profound vision loss is not completely understood, but one model proposes that activities in regions of the visual cortex immediately downstream of area V1 form the neural correlate of visual awareness^90^.

**Table 2:** The most common disorders of visual perception.

	Topological			Hodological		
	Cortex	Hyperfunction	Deficit	white matter	Hypoconnection	Hyperconnection
Subcortical		Phosphenes	Brainstem/subcortical visual deficits	Subcortical pathways	Subcortical pathways	Dazzle
V1/V2/V3	BA17/18	Phosphenes/simple hallucinations Visual snow syndrome Teichopsia	Phosphenes/simple hallucinations Visual snow syndrome Teichopsia	U-shaped	Cortical blindness*	
Depth (stereopsis)				Splenium	Astereopsis (split brain)	
Color	BA18/19	Color hallucination/illusion/hyperchromatopsia	Achromatopsia	Achromatopsia ILF§§/arcuate U-shaped	Color anomia*	Colored hearing, colored music, color grapheme synesthesia
Facial gestalt	BA37	Face hallucination/illusion Facial intermetamorphosis	Prosopagnosia*	ILF/IFOF	Prosopagnosia*	Pareidolia for faces
Facial features	BA19	Prosopometamorphopsia	Prosopagnosia*	ILF/IFOF	Prosopagnosia*	
Objects	BA 18/19/37	Object hallucination/illusion Lilliputian hallucinations	Object agnosia*/micropsia	ILF/IFOF/arcuate	Object agnosia*	Pareidolia for objects
Places	Parahippocampal gyrus	Landscape hallucinations	Environmental agnosia*			
Constancy		Micropsia/macropsia* Pelopsia/telopsia*	Micropsia/macropsia* Pelopsia/telopsia*		Micropsia/macropsia* Pelopsia/telopsia*	Micropsia/macropsia* Pelopsia/telopsia*
Spatialcoordinate frames	BA7/31	Polyopia/entomopia Visual perseveration Trailing phenomenon Delayed palinopsia Illusory visual spread Positive after image		U-shaped		Number forms

Abbreviations: BA, Brodmann area; ILF, Inferior longitudinal fasciculus; IFOF, Inferior fronto-occipital fasciculus;

* disorders likely to relate to both hodological and topological dysfunctions.

Source: Adapted from Ffytche and Howard^81^.

In conclusion, practicing neurologists encounter significant obstacles in the proper and on-time diagnosis to best care for patients with visual processing abnormalities. In these situations, anterior visual pathway assessments (visual field and acuity, fundoscopy, and pupillary) could show up as normal during an examination but will not explain the patient's specific visual complaints. A comprehensive evaluation of higher visual functions will be useful in localizing specific cortical visual disorders. The examination of these challenging cases offers a crucial foundation for thinking about significant neuroscientific theories about the way the brain functions.
